# Preoperative Prediction of the Aggressiveness of Oral Tongue Squamous Cell Carcinoma with Quantitative Parameters from Dual-Energy Computed Tomography

**DOI:** 10.3389/fonc.2022.904471

**Published:** 2022-06-23

**Authors:** Xieqing Yang, Huijun Hu, Fang Zhang, Dongye Li, Zehong Yang, Guangzi Shi, Guoxiong Lu, Yusong Jiang, Lingjie Yang, Yu Wang, Xiaohui Duan, Jun Shen

**Affiliations:** ^1^ Department of Radiology, Sun Yat-Sen Memorial Hospital, Sun Yat-Sen University, Guangzhou, China; ^2^ Guangdong Provincial Key Laboratory of Malignant Tumor Epigenetics and Gene Regulation, Medical Research Center, Sun Yat-Sen Memorial Hospital, Sun Yat-Sen University, Guangzhou, China

**Keywords:** Oral tongue squamous cell carcinoma, Dual-energy CT, Aggressiveness, pathologic stages, histologic differentiation, lymph node status

## Abstract

**Objectives:**

To determine whether quantitative parameters derived from dual-energy computed tomography (DECT) were predictive of the aggressiveness of oral tongue squamous cell carcinoma (OTSCC) including the pathologic stages, histologic differentiation, lymph node status, and perineural invasion (PNI).

**Methods:**

Between August 2019 and March 2021, 93 patients (mean age, 54.6 ± 13.8 years; 66 men) with pathologically diagnosed OTSCC were enrolled in this prospective study. Preoperative DECT was performed and quantitative parameters (e.g., slope of the spectral Hounsfield unit curve [λ_Hu_], normalized iodine concentration [nIC], normalized effective atomic number [nZ_eff_], and normalized electron density [nRho]) were measured on arterial phase (AP) and venous phase (VP) DECT imaging. Quantitative parameters from DECT were compared between patients with different pathologic stages, histologic differentiation, lymph node statuses, and perineural invasion statuses. Logistic regression analysis was utilized to assess independent parameters and the diagnostic performance was analyzed by the receiver operating characteristic curves (ROC).

**Results:**

λ_Hu_ and nIC in AP and λ_Hu_, nZ_eff_, and nIC in VP were significantly lower in stage III–IV lesions than in stage I–II lesions (*p* < 0.001 to 0.024). λ_Hu_ in VP was an independent predictor of tumor stage with an odds ratio (OR) of 0.29, and area under the curve (AUC) of 0.80. λ_Hu_ and nIC were higher in well-differentiated lesions than in poorly differentiated lesions (*p* < 0.001 to 0.021). The nIC in VP was an independent predictor of histologic differentiation with OR of 0.31, and AUC of 0.78. λ_Hu_ and nIC in VP were lower in OTSCCs with lymph node metastasis than those without metastasis (*p* < 0.001 to 0.005). λ_Hu_ in VP was the independent predictor of lymph node status with OR of 0.42, and AUC of 0.74. No significant difference was found between OTSCCs without PNI and those with PNI in terms of the quantitative DECT parameters.

**Conclusion:**

DECT can be a complementary means for the preoperative prediction of the aggressiveness of OTSCC.

## Introduction

Oral tongue squamous cell carcinoma (OTSCC) is the most common subtype of oral squamous cell carcinoma (OSCC), with 53,260 new cases in 2020 according to the Surveillance, Epidemiology, and End Results Program (1, 2). It is known that the OTSCC differs from other OSCC in the epithelial origin, lymphatic drainage, aggressiveness, and prognosis (3, 4). The OSCCs originating from different types of oral epithelia have different tumor characteristics, recurrence and survival rates, and responsiveness to the treatment modalities (3). For example, the OTSCC is more likely to metastasize to cervical lymph nodes than other OSCCs from the hard palate or upper gum (4). Surgical resection is a primary treatment modality for OTSCC (5). Postoperative recurrence remains a great challenge in patients with OTSCC (6, 7). Several clinicopathological features including pathological stage, histologic differentiation, lymph node metastasis, and perineural invasion (PNI), which were considered reliable markers for biological aggressiveness of OTSCC, have been redeemed also as important risk factors associated with carcinoma recurrence and prognosis of OTSCC patients following surgical resection (8–11). However, pathological stage, histologic differentiation, lymph node metastasis, and PNI are determined histologically after resection. The postoperative pathological assessment may result in overtreatment or undertreatment. For example, elective neck dissection (END) for N0 tongue cancer might thus result in unnecessarily invasive procedures in at least 60% of patients (12). On the other hand, inadequate treatment that underestimates tumor aggressiveness such as clinical stage may expose OTSCC patients to require secondary surgical intervention or increase the risk of recurrence. Accurate and comprehensive evaluation of OTSCC aggressiveness before the operation, therefore, is critical for generating an effective and individualized treatment plan for OTSCC patients.

Computed tomography (CT) and magnetic resonance imaging (MRI) are the most recommended and widely used imaging modalities for noninvasive and preoperative assessment of the aggressive features of OTSCC (13–17). Previously, dynamic contrast-enhanced MRI (DCE-MRI) and diffusion kurtosis imaging (DKI) has been reported to be able to evaluate the pathologic stage (15), histologic differentiation, and lymph node metastasis in oral carcinoma (18). However, some patients cannot undergo MRI examination because of certain contraindications such as claustrophobia and pacemakers. Some people cannot do contrast enhanced MRI due to renal function and risk for nephrogenic fibrosis. Moreover, different protocols, variabilities in analysis procedures, and image artifacts of functional MRI influence the accuracy and repeatability. Conventional CT can rely on morphological information to partially assess tumor extension (14, 19, 20) and lymph node metastasis (21). However, the morphologic criterion of cervical node metastasis in conventional CT cannot accurately assess the status of cervical nodes without sufficient specificity (21). Moreover, functional parameters derived from conventional CT with better diagnostic performance in the preoperative assessment of OTSCC aggressiveness are lacking. Dual-energy CT (DECT) can acquire information simultaneously at two different peak energies, enabling the differentiation of materials with varying molecular components based on their photoelectric absorption profiles (22). It can reflect histological and hemodynamic information for tissue characterization by providing multiple quantitative parameters (e.g., slope of the spectral Hounsfield unit curve [λ_Hu_], effective atomic number [Z_eff_], electron density [Rho], iodine concentration [IC], and normalized iodine concentration [nIC]) (23–26).

Previously, quantitative parameters derived from DECT have been reported to be able to effectively assess the aggressiveness of lung cancer (27), renal cell carcinoma (28), and colon cancer (29). DECT has been also determined as a useful tool for improving diagnosis and nodal staging as well as for evaluating the invasion of critical structures such as the thyroid cartilage in head and neck squamous cell carcinoma (22). However, whether quantitative parameters from DECT can be used to assess OTSCC aggressiveness preoperatively, including pathologic TNM stages, histologic differentiation, lymph node metastasis, and PNI, remains unknown.

We hypothesized that quantitative parameters from DECT can assess OTSCC aggressiveness. In this study, DECT was performed in OTSCC patients, and quantitative parameters of primary lesions were obtained. This study aimed to determine whether quantitative parameters derived from DECT were predictive of the aggressiveness of OTSCC.

## Materials and Methods

### Patients

**Figure 1:**
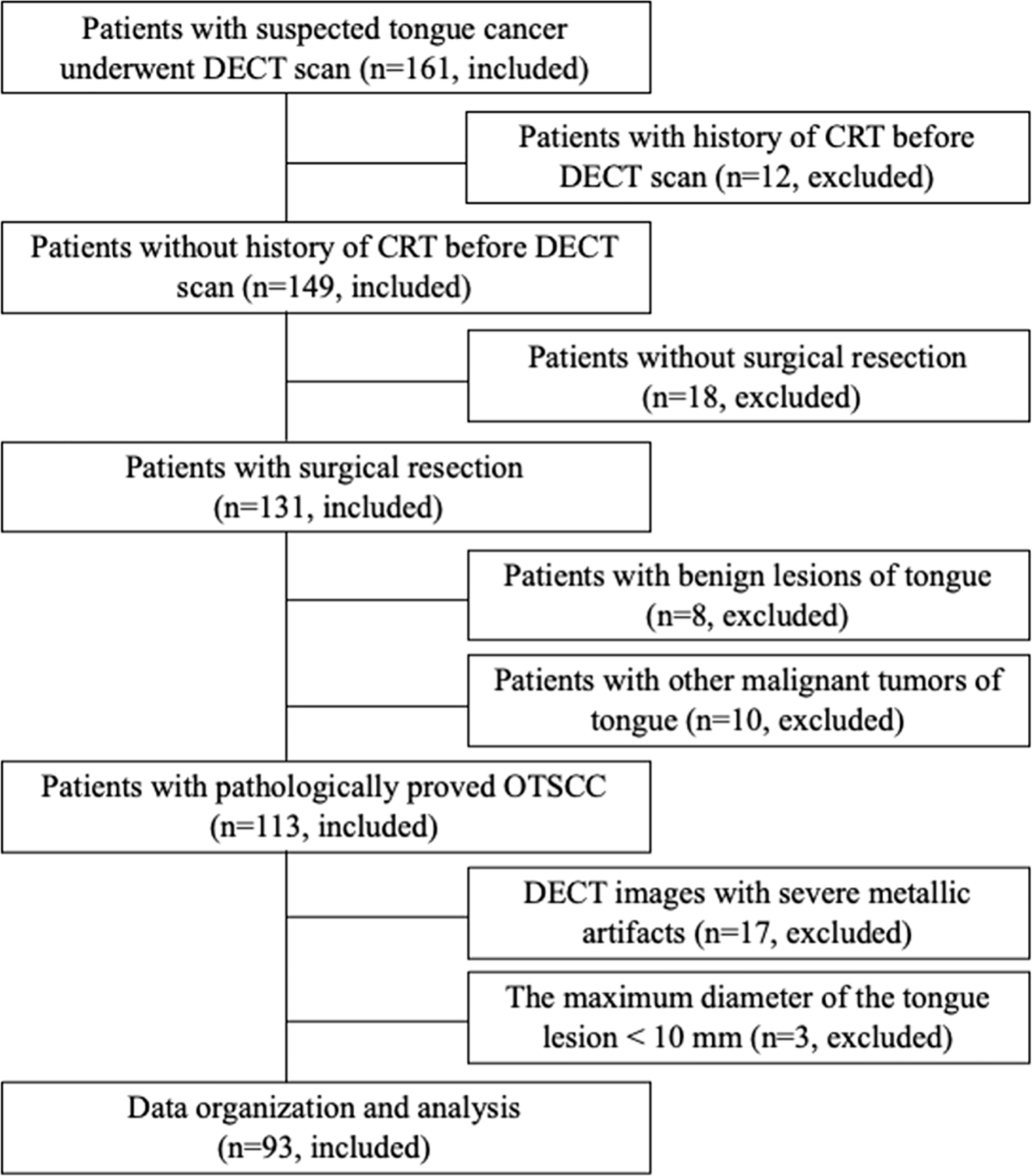
Flowchart shows the strategy for OTSCC analysis and data processing in this study.

The ethics committee of our hospital approved this prospective, single-center study, and written informed consent was obtained from all participants. From August 2019 to March 2021, 161 consecutive patients who were suspected of having tongue cancer on physical examination were enrolled and underwent DECT examination. Ninety-three patients with pathologically confirmed OTSCC and who underwent surgical resection (<2 weeks interval between surgical resection and DECT examination) were included. The exclusion criteria were as follows: 1) previous history of chemotherapy and/or radiation therapy; 2) no surgical resection; 3) >2 weeks of interval between surgical resection and DECT examination; 4) evident metallic artifacts; and 5) the maximum dimension of the tongue lesion measured on DECT images was less than 10 mm. The flow chart of patient enrollment is shown in **Figure 1**. The demographic and clinicopathological characteristics of these patients are shown in **Table 1**.

**Table 1:** The clinicopathologic characteristics of patients with OTSCC (n = 93).

Characteristics	Mean ± SD or n (%)
Age, years	54.6 ± 13.8 (26-84)
Gender, male	66 (71.0)
TNM stage
T stage
1	11 (11.8)
2	41 (44.1)
3	26 (28.0)
4	15 (16.1)
N stage
0	48 (51.6)
1	17 (18.3)
2	23 (24.7)
3	5 (5.4)
M stage
0	93 (100.0)
1	0 (0.0)
Overall pathologic stage
Stage I	11 (11.8)
Stage II	30 (32.3)
Stage III	20 (21.5)
Stage IV	32 (34.4)
Histologic differentiation
Poorly differentiated	29 (31.2)
Moderately differentiated	42 (45.1)
Highly differentiated	22 (23.7)
Lymph node metastasis
Absent	48 (51.6)
Present	45 (48.4)
Perineural invasion
Absent	65 (69.9)
Present	28 (30.1)

OTSCC, Oral tongue squamous cell carcinoma.

### DECT Imaging

All participants underwent head and neck DECT examination within 2 weeks before the surgery on 96 multi-detector row CT scanner (SOMATOM Force, Siemens Healthcare). After patients were injected with a nonionic iodinated contrast medium (350 mg I/mL iohexol, GE Healthcare) at a dose of 1.0 mL/kg using a power syringe (Bayer Medical Care Inc., Indianola, PA, USA) at a flow rate of 4 mL/s *via* the anterior cubital vein, dual-phase contrasted-enhanced CT images were achieved in the DECT scan mode with the scanning parameters as follows: tube voltage and current (tube A, 70 kVp and 250 mAs; tube B Sn, 150 kVp and 71 mAs); pitch, 0.5; gantry rotation time, 0.25 s; and section collimation, 128×0.6 mm. The scanning delay for arterial phase (AP) imaging was determined with using a bolus tracking triggering software. Arterial phase scanning automatically began 3 s after the trigger attenuation threshold (100 HU) was reached at the level of the internal carotid artery. Venous phase (VP) scanning was performed at a delay of 20 s after AP scanning. DECT datasets were reconstructed into monochromatic image (photon energies, 40 ~ 190 keV) and iodine maps with a section thickness of 1.0 mm and an increment of 0.7 mm, an FOV of 250 mm, and a matrix of 512 × 512 (30). CT data of 93 OTSCC participants were reconstructed by use of Advanced Modeled Iterative Reconstruction (ADMIRE, Siemens Healthineers) with a strength of 3.

### Image Analysis

DECT data analysis were performed on the viewer software at the workstation (Syngo.via, Siemens Healthcare, Erlangen, Germany). Quantitative DECT parameters of primary tumors were assessed independently by two experienced radiologists (X.Y. and F.Z. with 3 and 12 years of experience in head and neck radiology, respectively) who were blinded to the histopathologic results and clinical information of 93 OTSCC participants. Three consecutive image sections comprising the largest section of the lesion on axial images and the upper and lower levels were chosen for measurement. A free-hand region of interest (ROI) was drawn to cover the entire tumor with care to exclude large feeding vessels and necrotic areas. For each patient, ROI was separately outlined on each of three image sections and the ROI-based values were averaged. Quantitative DECT parameters measured by X.Y. and F.Z. were averaged to obtain the values for final analysis. CT values of primary tumors were measured on monochromatic images (Hounsfield unit [HU]). IC was derived from iodine maps in milligrams per cubed centimeter; Z_eff_ and Rho were automatically calculated by workstation software. The IC, Z_eff_ and Rho of primary tumor was normalized to those of the same side of the carotid artery to obtain normalized IC (nIC), Z_eff_ (nZ_eff_) and Rho (nRho). The slope of the attenuation curve, λ_Hu_ (in HU per kiloelectron-volt) is calculated as the difference between the CT value at 40 keV and that at 70 keV divided by the energy difference (30 keV) according the formula λ_Hu_ = (HU_40keV_−HU_70keV_)/30 keV, where *HU_40keV_* refers to the CT value measured on 40-keV images and *HU_70keV_* represents the CT value measured on 70-keV images (30). The quantitative DECT parameters of the primary lesions were obtained from both AP and VP contrast-enhanced images. One of the two radiologists (X.Y.) repeated the measurement of quantitative DECT parameters of all primary lesions 2 months later to determine the intra-observer reproducibility. A time interval of 2 months was set to minimize recall bias.

### Surgery and Histology

All participants underwent surgical resection of the primary tongue tumor and neck dissection resection. The whole resected tissue samples were processed for conventional histological analysis. Pathologic characteristics of OTSCC, i.e., pathologic TNM stages, histologic differentiation, lymph node status, and PNI were recorded. Cervical lymph node status was determined from the neck dissection resection. TNM staging was graded according to the 8th American Joint Committee on Cancer (AJCC) staging system, and the overall pathologic stages of OTSCCs are classified into two stages: early (stages I–II) and late stage (stages III–IV) (31). The presence of PNI and tumor differentiation were determined according to the grading system of the World Health Organization (32). The tumor histologic differentiation of OTSCCs is divided into well-differentiated (moderately or highly differentiated) and poorly differentiated differentiation.

### Statistical Analysis

All quantitative variables were presented as mean ± standard deviation (SD), and categorical variables were described as frequency. Inter- and intra-observer agreements of the evaluation of quantitative DECT parameters were assessed using the intra-class correlation coefficient (ICC). The strength of the agreement was rated as follows: <0.40, poor agreement; 0.40–0.59, fair agreement; 0.60–0.74, good agreement; and 0.75–1.0, excellent agreement. Quantitative DECT parameters were compared among OTSCC patients with different aggressiveness using Student’s *t*-test when F‐test validated homogeneity of variance. Otherwise, the non-parametric Mann-Whitney U-test was used. Univariable logistic regression analysis was utilized to determine if a single parameter was associated with the stages, histologic differentiation, lymph node status, and PNI of OTSCC. Multicollinearity analysis was performed to test the independence of predictive parameters using variance inflation factors (VIF) and tolerance. VIF < 10 and tolerance > 0.1 were set as no multicollinearity. Multivariable binary logistic regression analysis was used to determine the independent parameters for predicting the stages, histologic differentiation, lymph node status, and PNI of OTSCC. Odds ratios (OR) with 95% confidence intervals (CI) were obtained for each independent predictor. Receiver operating characteristic (ROC) analysis was utilized to evaluate the diagnostic ability of significant parameters. The area under the curve (AUC), sensitivity, specificity, and accuracy were computed for each significant parameter, and the optimal threshold was determined by the Youden index. ROC analysis was using by the glmnet, pROC, survival, nircens, and ggplot2 packages of R software (version 3.5.0, R Project). Other statistical analyses were processed with SPSS software (version 23.0, IBM Corp.). A two-sided *p* < 0.05 was set to indicate significance.

## Results

### Study Population

Of the 161 patients with suspected tongue cancer enrolled in this study, 68 were excluded because they received chemotherapy and/or radiation therapy before CT (n = 12), did not undergo surgical resection (n =18), had benign tongue lesions confirmed by biopsy pathology (n = 8), had other malignant tumors of the tongue (n = 10), had severe metallic artifacts in DECT images (n = 17), and had a maximum dimension of the tongue lesion < 10 mm (n = 3). Finally, 93 OTSCC participants were included in our study. Of the 93 patients, the mean age was 54.6 ± 13.8 (age range, 26–84) years, 71.0% (66/93) were men, and 29.0% (27/93) were women. Overall, 93 patients had 93 tongue tumors. The clinical features of 93 OTSCC participants are summarized in **Table 1**. Of 93 OTSCC lesions, 41 were in the early-stage (stages I–II) and 52 in the advanced-stage (stages III–IV); 64 were well-differentiated (moderately or highly differentiated) and 29 were poorly differentiated. In addition, 45 patients had lymph node metastasis and 48 patients had no metastasis; 28 patients had PNI, and 65 patients had no PNI.

### Quantitative DECT Parameters and OTSCC Aggressiveness

The ICC analysis indicated good concordance inter- and intra-observer agreements (ICC, 0.929–0.987, 0.967–0.987, respectively; **Table S1**). The average values of the quantitative DECT parameters between the different groups stratified by pathologic stages, histologic differentiation, lymph node status, and PNI, are shown in **Table 2**. The mean λ_Hu_ and nIC in AP and λ_Hu_, nZ_eff_, and nIC in VP of stage I–II OTSCCs were higher than those of stage III–IV OTSCCs (*p* < 0.001 to 0.024, **Table 2**), while the mean nZ_eff_ in AP and nRho both in AP and VP were not significantly different between stage I–II and stage III–IV OTSCCs. Univariate logistic regression analysis showed that the mean λ_Hu_ and nIC in AP and λ_Hu_, nZ_eff_, and nIC in VP were associated with the tumor stage (*p* < 0.001 to 0.047, **Table 3**). No multicollinearity was noted in the quantitative DECT parameters (VIF < 10 and tolerance > 0.1). On the grounds of multivariable logistic regression analysis, λ_Hu_ in VP was the independent predictor of tumor stage with OR of 0.29 (95% CI, 0.16–0.52, *p* < 0.001, **Table 3**).

**Table 2:** Quantitative DECT parameters between OTSCC patients with different aggressiveness (n = 93).

Parameters	Pathologic stages			Histologic differentiation			Lymph node status of OTSCC			PNI status of OTSCC		
	Stage I–II	Stage III-IV	*p* value	Well-differentiated	Poorly differentiated	*p* value	Without metastasis	With metastasis	*p* value	Without PNI	With PNI	*p* value
λ_Hu_ in AP^a^	2.30 ± 0.68	1.87 ± 0.63	0.003	2.20 ± 0.72	1.74 ± 0.46	0.003	2.19 ± 0.71	1.92 ± 0.63	0.058	2.06 ± 0.69	2.04 ± 0.68	0.980
nIC in AP^a^	0.17 ± 0.07	0.14 ± 0.06	0.014	0.16 ± 0.07	0.13 ± 0.06	0.021	0.16 ± 0.07	0.15 ± 0.06	0.154	0.15 ± 0.07	0.16 ± 0.07	0.808
nZ_eff_ in AP^b^	0.72 ± 0.05	0.71 ± 0.05	0.699	0.72 ± 0.04	0.71 ± 0.06	0.923	0.72 ± 0.05	0.71 ± 0.05	0.990	0.71 ± 0.05	0.72 ± 0.04	0.507
nRho in AP^b^	0.67 ± 0.15	0.67 ± 0.12	0.896	0.67 ± 0.14	0.67 ± 0.12	0.986	0.67 ± 0.14	0.67 ± 0.12	0.753	0.68 ± 0.15	0.65 ± 0.10	0.290
λ_Hu_ in VP^b^	3.43 ± 0.76	2.67 ± 0.65	<0.001	3.22 ± 0.83	2.52 ± 0.43	<0.001	3.29 ± 0.81	2.70 ± 0.68	<0.001	3.09 ± 0.86	2.81 ± 0.60	0.132
nIC in VP^b^	0.57 ± 0.13	0.45 ± 0.13	<0.001	0.55 ± 0.14	0.41 ± 0.09	<0.001	0.54 ± 0.14	0.46 ± 0.14	0.005	0.51 ± 0.15	0.48 ± 0.13	0.245
nZ_eff_ in VP^a^	0.89 ± 0.05	0.86 ± 0.06	0.024	0.87 ± 0.06	0.87 ± 0.04	0.274	0.88 ± 0.05	0.86 ± 0.06	0.081	0.87 ± 0.05	0.87 ± 0.06	0.847
nRho in VP^b^	0.83 ± 0.17	0.83 ± 0.14	0.998	0.84 ± 0.17	0.82 ± 0.13	0.589	0.84 ± 0.16	0.83 ± 0.15	0.934	0.83 ± 0.16	0.85 ± 0.14	0.622

^a^Mann–Whitney U test; ^b^Student’s t-test; DECT, Dual-energy computed tomography; OTSCC, Oral tongue squamous cell carcinoma; PNI, perineural invasion; λ_Hu_, slope of the spectral Hounsfield unit curve; nIC, normalized iodine concentration; nZ_eff_, normalized effective atomic number; nRho, normalized electron density; AP, arterial phase; VP, venous phase.

**Table 3:** Univariate and multivariate logistic analyses of quantitative DECT parameters in predicting the aggressiveness of OTSCC.

Parameters	Pathologic stages				Histologic differentiation				Lymph node status			
	Univariate regression		Multivariate regression		Univariate regression		Multivariate regression		Univariate regression		Multivariate regression	
	OR (95%CI)	*P* value	OR (95%CI)	*P* value	OR (95%CI)	*P* value	OR (95%CI)	*P* value	OR (95%CI)	*P* value	OR (95%CI)	*P* value
λ_Hu_ in AP	0.51 (0.32,0.81)	0.004		0.146	0.45 (0.26,0.78)	0.004		0.849	0.66 (0.43,1.02)	0.060		
nIC in AP	0.63 (0.40,0.98)	0.039		0.683	0.59 (0.35,1.00)	0.049		0.237	0.77 (0.50,1.16)	0.202		
nZ_eff_ in AP	0.92 (0.61,1.39)	0.695			0.98 (0.63,1.52)	0.922			1.00 (0.66,1.50)	0.990		
nRho in AP	1.03 (0.68,1.56)	0.895			1.00 (0.64,1.55)	0.986			1.07 (0.71,1.61)	0.750		
λ_Hu_ in VP	0.29 (0.16,0.52)	<0.001	0.29 (0.16,0.52)	<0.001	0.27 (0.13,0.54)	<0.001		0.098	0.42 (0.25,0.70)	0.001	0.42 (0.25,0.70)	0.001
nIC in VP	0.39 (0.24,0.63)	<0.001		0.414	0.31 (0.17,0.55)	<0.001	0.31 (0.17,0.55)	<0.001	0.54 (0.35,0.85)	0.007		0.905
nZ_eff_ in VP	0.62 (0.39,0.99)	0.047		0.058	0.91 (0.59,1.41)	0.683			0.69 (0.45,1.07)	0.099		
nRho in VP	1.00 (0.66,1.51)	0.998			0.88 (0.57,1.38)	0.585			0.98 (0.65,1.45)	0.933		

DECT, Dual-energy computed tomography; OTSCC, Oral tongue squamous cell carcinoma; OR, odds ratio; CI, confidential interval; λ_Hu_, slope of the spectral Hounsfield unit curve; nIC, normalized iodine concentration; nZ_eff_, normalized effective atomic number; nRho, normalized electron density; AP, arterial phase; VP, venous phase.

λ_Hu_ and nIC in AP and λ_Hu_, and nIC in VP of well-differentiated OTSCCs were higher than those of poorly differentiated OTSCCs (*p* < 0.001 to 0.021, **Table 2**). nZ_eff_ and nRho both in AP and VP, were not different between well-differentiated OTSCCs and poorly differentiated OTSCCs. From univariable logistic regression analysis, the mean λ_Hu_ and nIC in AP and λ_Hu_, and nIC in VP were associated with the degree of differentiation of OTSCCs (*p* < 0.001 to 0.049, **Table 3**). Multivariable logistic regression analysis showed that the nIC in VP was an independent predictor of histologic differentiation of OTSCC with OR of 0.31 (95% CI, 0.17–0.55, *p* < 0.001, **Table 3**).

The mean λ_Hu_ and nIC in VP were lower in OTSCCs with lymph node metastasis compared with those of OTSCCs without metastasis (*p* < 0.001 to 0.005, **Table 2**). λ_Hu_, nIC, nZ_eff_, and nRho in AP and nZ_eff_ and nRho in VP were not significantly different between OTSCCs with lymph node metastasis than those of OTSCCs without metastasis. From univariable logistic regression analysis, the mean λ_Hu_ and nIC in VP were associated with lymph node status in OTSCC (*p* < 0.001 to 0.007, **Table 3**). The multivariable logistic regression analysis indicated that λ_Hu_ in VP was the independent predictor of the lymph node status of OTSCC with OR of 0.42 (95% CI, 0.25–0.70, *p* < 0.001, **Table 3**).

No significant difference was observed between the group without PNI and the group with PNI in terms of the quantitative DECT parameters (*p* = 0.132 to 0.980, **Table 3**).

### Predictive Performance of Quantitative DECT Parameters

**Figure 2:**
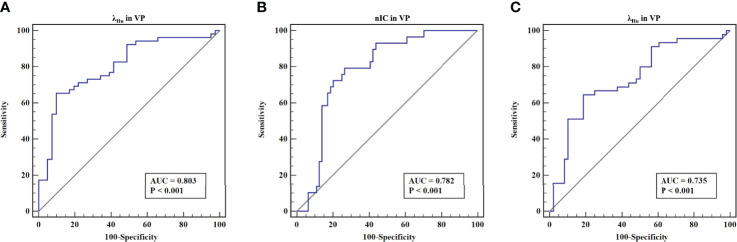
The receiver operating characteristic curves of quantitative DECT parameters for discriminating the different aggressiveness of OTSCC. **(A)** The receiver operating characteristic curves of λ_Hu_ in VP for discriminating stage III-IV lesions from stage I-II lesions. **(B)** The receiver operating characteristic curves of nIC in VP for discriminating well-differentiated lesions (moderately or highly differentiated) from poorly differentiated lesions. **(C)** The receiver operating characteristic curves of λ_Hu_ in VP for discriminating lesions without lymph node metastasis from those with lymph node metastasis.

**Figure 3:**
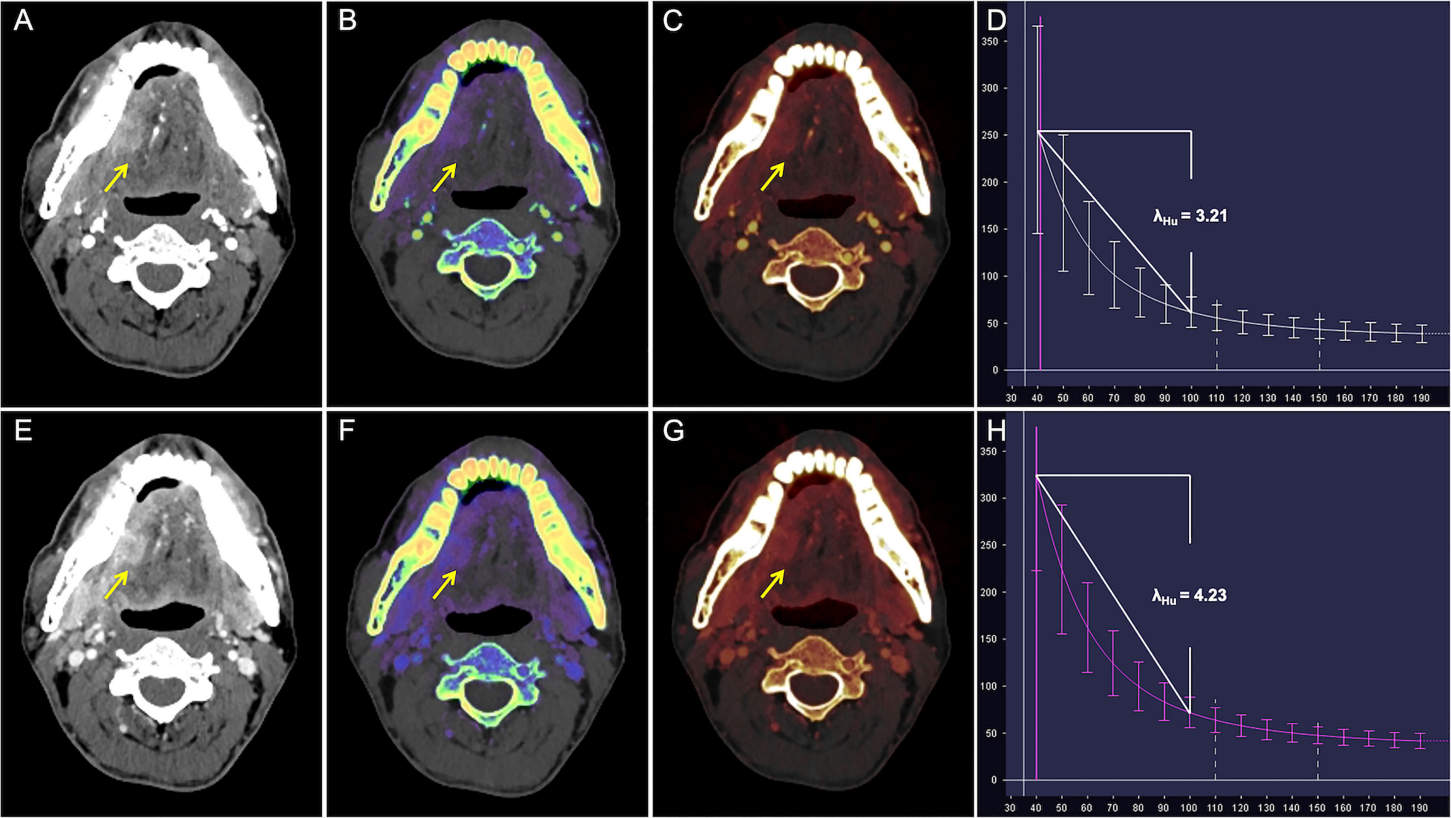
CT images in a 50-year-old man with well-differentiated T2N0M0 OTSCC in stage II. **(A–D)** Arterial phase imaging of the primary tumor (arrow). **(A)** Contrast-enhanced 40-keV monochromatic image shows lesion with mean CT value of 255.26 HU. **(B)** Effective atomic number map shows lesion with mean Z_eff_ value of 8.70. **(C)** Iodine-based pseudo-colorized image shows lesion with mean IC of 3.16 mg/ml. **(D)** Spectral Hounsfield unit curve shows lesion with mean λ_Hu_ of 3.21 HU/keV. **(E–H)** Venous phase imaging of the primary tumor (arrow). **(E)** Contrast-enhanced 40-keV monochromatic image shows lesion with mean CT value of 325.53 HU. **(F)** Effective atomic number map shows lesion with mean Z_eff_ value of 9.17. **(G)** Iodine-based pseudo-colorized image shows lesion with mean IC of 3.50 mg/ml. **(H)** Spectral Hounsfield unit curve shows lesion with mean λ_Hu_ of 4.23 HU/keV.

**Figure 4:**
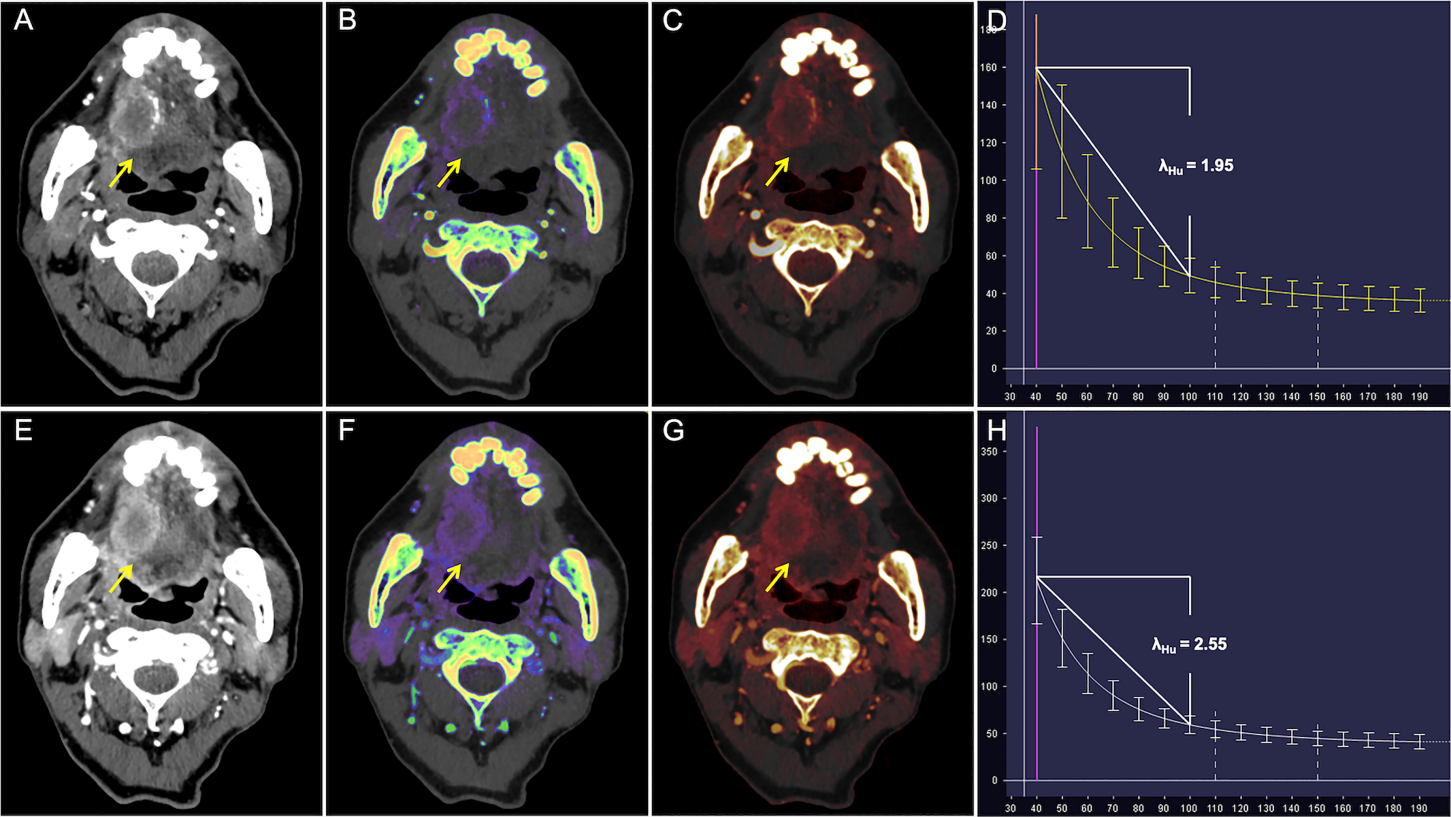
CT Images in a 65-year-old man with poorly differentiated T3N1M0 OTSCC in stage III. **(A–D)** Arterial phase imaging of the primary tumor (arrow). **(A)** Contrast-enhanced 40-keV monochromatic image shows lesion with mean CT value of 167.12 HU. **(B)** Effective atomic number map shows lesion with mean Z_eff_ value of 8.37. **(C)** Iodine-based pseudo-colorized image shows lesion with mean IC of 1.53 mg/ml. **(D)** Spectral Hounsfield unit curve shows lesion with mean λ_Hu_ of 1.95 HU/keV. **(E–H)** Venous phase imaging of the primary tumor (arrow). **(E)** Contrast-enhanced 40-keV monochromatic image shows lesion with mean CT value of 212.34 HU. **(F)** Effective atomic number map shows lesion with mean Z_eff_ value of 8.72. **(G)** Iodine-based pseudo-colorized image shows lesion with mean IC of 2.2 mg/ml. **(H)** Spectral Hounsfield unit curve shows lesion with mean λ_Hu_ of 2.55 HU/keV.

ROC analysis of quantitative DECT parameters predictive of the overall pathologic stage in OTSCC is shown in **Table 4** and **Figure 2A**. In predicting the overall pathologic stage, the λ_Hu_ in VP had the highest AUC (0.80, 95% CI, 0.71–0.89) with a sensitivity of 65.4%, a specificity of 87.8% and an accuracy of 75.3%. The diagnostic performances of the quantitative DECT parameters for predicting the histologic differentiation of OTSCC are shown in **Table 4** and **Figure 2B**. In predicting the histologic differentiation of OTSCC, the nIC in VP had the highest AUC (0.78, 95% CI, 0.69–0.88) with a sensitivity of 75.9%, a specificity of 75% and an accuracy of 75.3% (**Table 4**; **Figure 2B**). The results of the ROC analysis for predicting the lymph node metastasis of OTSCC are summarized in **Table 4** and **Figure 2C**. In predicting the lymph node metastasis of OTSCC, the λ_Hu_ in VP had the highest AUC (0.74, 95% CI, 0.63–0.84) with a sensitivity of 64.4%, a specificity of 79.2% and an accuracy of 72.0% (**Table 4**; **Figure 2C**). Two example cases of a well-differentiated OTSCC of T2N0M0 (stage II) and a poorly differentiated OTSCC of T3N1M0 (stage III) are shown in **Figures 3** and **4**.

**Table 4:** Receiver operating characteristic curve analysis of quantitative DECT parameters for discriminating the different aggressiveness of OTSCC.

Parameters	AUC (95%CI)	Threshold	Sensitivity (95%CI)	Specificity (95%CI)	Accuracy (%)
**Pathologic stages**					
λ_Hu_ in VP	0.80 (0.71,0.89)	2.66	65.4 (50.9,78.0)	87.8 (73.8,95.9)	75.3 (65.2,83.6)
**Histologic differentiation**					
nIC in VP	0.78 (0.69,0.88)	0.46	75.9 (56.5,89.7)	75.0 (62.6,85.0)	75.3 (65.2,83.6)
**Lymph node status**					
λ_Hu_ in VP	0.74 (0.63,0.84)	2.66	64.4 (48.8,78.1)	79.2 (65.0,89.5)	72.0 (61.8,80.9)

DECT, Dual-energy computed tomography; OTSCC, Oral tongue squamous cell carcinoma; AUC, area under curves; CI, confidential interval; λ_Hu_, slope of the spectral Hounsfield unit curve; nIC, normalized iodine concentration; VP, venous phase.

## Discussion

Our study results showed that several quantitative DECT parameters differed in OTSCC lesions with different pathologic TNM stages, histologic differentiation, and lymph node metastasis of OTSCC. Certain quantitative DECT parameters showed high performance in predicting OTSCC aggressiveness, and the parameters obtained VP had higher diagnostic ability than in those obtained in AP.

OTSCC shows heterogeneity in terms of patient characteristics, histological subtypes, stages, and treatment outcomes, which can be characterized by image analysis on the basis of quantitative image features (33). Previously, the stage III-IV OTSCCs were found to have lower values of kinetic parameters (i.e., K^trans^, K_ep_ and V_p_ values) compared with the early stage I-II OTSCCs; and the K_ep_ had the highest predictive performance for discriminating between the advanced-stage OTSCCs and early-stage OTSCCs with an AUC of 0.731 (15). In addition, quantitative DKI parameters (diffusivity and kurtosis) were found to be associated with the histologic differentiation grades of oral cancer (18); and the AUCs of diffusivity and kurtosis were 0.989 and 0.978 in identifying the poorly or moderately differentiated lesions from well-differentiated lesions, respectively (18). In this study, DECT was used to assess the aggressiveness of OTSCC. To the best of our knowledge, this is the first prospective study to evaluate the association between quantitative DECT parameters and OTSCC aggressiveness.

Our study showed that advanced-stage OTSCCs had lower mean λ_Hu_ and nIC in AP and λ_Hu_, nZ_eff_, and nIC in VP than early-stage OTSCCs. λ_Hu_ in VP was an independent predictor of tumor stage. The λ_Hu_ in VP threshold of 2.66 optimizes the discrimination between advanced-stage OTSCCs and early-stage OTSCCs with AUC of 0.80. The slope value derived from the spectral attenuation curve can be utilized to quantify the iodine (CT contrast) content in different tissues (23). Different substances have unique characteristic curves and λ_Hu_. IC from DECT had well agreement with the actual iodine amount, which served as a quantitative biomarker of blood volume in the tissues (24–26). nIC refers to the IC value standardized using the carotid artery, which has been used to minimize the variations in circulation status and scanning time among patients (30). Z_eff_, the mean atomic number of mixture substances in tissue, was correlated with the density of cellular components and iodine content within carcinomas (23). These parameters are associated with iodine content. In this study, advanced-stage OTSCCs had lower λ_Hu_, nIC, and nZ_eff_ than the early-staged OTSCCs. The possible reason was that the larger the hypoxic areas, the less vessel density was in more invasive OSCCs (34), which led to lower iodine content into the tumor, resulting in lower λ_Hu_, nIC, and nZ_eff_. In a previous study, Guo et al. (15) found that advanced-stage OTSCCs have less vessel density and vascular permeability in DCE-MRI, which corresponded with our results. On the other hand, Yu et al. found that advanced-stage OSCCs had higher collagen content than early-stage OSCCs (35). Collagen accumulation can lead to the elevation of the interstitial fluid pressure (36), which is a crucial factor for the extravasation of iodine into tumor tissues. Therefore, collagen accumulation might be another explanation why advanced-stage OTSCCs had lower parameter values in our study.

Quantitative DECT parameters have been utilized for predicting the histologic differentiation in lung (27), renal (28), and colon cancers (29). Li et al. reported that poorly differentiated lung cancer had lower IC in VP than well-differentiated lung cancer (27). Wei et al. found that high-grade clear cell renal cell carcinoma (poorly differentiated) had lower λ_HU_ and nIC than low-grade clear cell renal cell carcinoma (well-differentiated) (28). By contrast, Yang et al. reported that poorly differentiated or undifferentiated colon cancer had higher λ_HU_, IC, and nIC in AP than well-differentiated colon cancer (29). Our results showed that poorly differentiated OTSCCs had lower λ_Hu_, and nIC than well-differentiated lesions. Previous studies suggested that the poorly differentiated OTSCCs possess a high degree of malignancy, more intensive cell proliferation, high HIF-1 alpha expression, and reduced vessel density (37–39). Insufficient vascularization and increased lesion size may cause inadequate blood supply and tumor necrosis. Hence, our study suggested that quantitative DECT parameters may be useful image biomarkers in the differential diagnosis of OTSCC with different degrees of differentiation.

Our results also showed that the λ_Hu_ in VP was an independent predictor of the lymph node status of OTSCC and had a moderate diagnostic ability with an AUC of 0.74. Uehara et al. showed that OTSCC patients with lymph node metastasis had high expression of HIF-1 alpha compared with those without metastasis (40). Moreover, the primary tumor necrosis in N2c and N3 stages was higher than those in N1, N2a, and N2b stages (41), which means that OTSCCs with lymph node metastasis had more highly hypoxic areas and poor blood supply than those without node metastasis. Therefore, these features can be possible explanations for the finding that OTSCC with nodal metastasis had lower quantitative DECT parameter values than those without metastasis.

In this study, ROC analysis revealed that quantitative DECT parameters had higher performance in VP than those in AP for predicting pathologic stages, histologic differentiation, and lymph node status. During contrast-enhanced CT, diffusion of the contrast agents in the tumor is mainly affected by neovascularization density and the amount of contrast leaking into the tumor interstitial space (42). In the AP, the contrast enhancement of primary OTSCC is mainly associated with the filling of contrast agents in the microvasculature, which is affected by the density of neovascularization and the disorder and tortuosity of the microvasculature in the tumor (27). As the disorder and tortuosity of the microvasculature and necrosis in OTSCC, the flow rate of contrast agents is often slow and poor in more aggressive OTSCCs (27). In VP, CT contrast agents enter the tumor interstitial space, and the contrast enhancement is primary associated with the retention of contrast agents in extravascular extracellular space (27). More predominant difference in extravascular extracellular space rather than in degree of functional neovascularization among OTSCCs with different aggressiveness could be the reason why quantitative DECT parameters in VP demonstrated better performance than those in AP. This phenomenon was also observed in lung cancer (27).

This study evaluated the associations between quantitative DECT parameters and OTSCC aggressiveness. The results show DECT were predictive of the aggressiveness of OTSCC based on quantitative parameters. Quantitative DECT parameters is lower in highly invasive than less invasive OTSCC. Therefore, the present study may provide an important basis for clinical individualized chemotherapies. For instance, an intensified multimodal therapy such as surgical resection of the primary lesion combined with END may be used for cT1-2N0 OTSCC instead of conservative clinical therapy.

This study had several limitations. First, potential bias was possible in this single-center study, which might be a reason for the inapparent difference in quantitative DECT parameters between OTSCCs with PNI and those without PNI in our study. Hence, large-sample, and multicenter studies are warranted. Second, quantitative parameters acquired on the CT section were barely matched with the corresponding histological slice. The inevitable mismatch between the histopathologic specimen and CT image would have influenced the degree of correlation. Third, although this study investigated the correlation between DECT parameters and OTSCC aggressiveness, including pathologic stages, histologic differentiation, lymph node status, and PNI, a more comprehensive evaluation of OTSCC aggressiveness such as extranodal extension using DECT should be explored in the future.

In summary, quantitative DECT parameters were risk factors and had a high performance for the preoperative prediction of OTSCC aggressiveness, including pathologic TNM stages, histologic differentiation, and lymph node status. DECT might be a useful tool for the preoperative evaluation of OTSCC aggressiveness, which could be valuable for deciding the treatment strategy and determining the prognosis of patients with OTSCC.

## Data Availability Statement

The original contributions presented in the study are included in the article/**Supplementary Material**. Further inquiries can be directed to the corresponding authors.

## Ethics Statement

The studies involving human participants were reviewed and approved by The ethics committee of Sun Yat-Sen Memorial Hospital (Sun Yat-Sen University, Guangzhou, China). The patients/participants provided their written informed consent to participate in this study. Written informed consent was obtained from the individual(s) for the publication of any potentially identifiable images or data included in this article.

## Author Contributions

XY and FZ contributed to the conception and design of the study. XY, HH, GS, GL, YJ, LY and YW collected the data. XY, FZ, DL, ZY, XD and JS analyzed and interpreted the data. XY and HH drafted the manuscript. JS and XD revised the manuscript. All authors contributed to the article and approved the submitted version.

## Funding

This study has received funding from the National Natural Science Foundation of China (No: U1801681, 82171996), the Key Areas Research and Development Program of Guangdong (2019B020235001) and Guangdong Province Universities and Colleges Pearl River Scholar Funded Scheme (2017), the Project Supported by GuangDong Basic and Applied Basic Research Foundation (2020), and the Medical Research Foundation of Guangdong Province of China (Grant No. 2020A1515110478) for sample collection and data acquisition.

## Conflict of Interest

The authors declare that the research was conducted in the absence of any commercial or financial relationships that could be construed as a potential conflict of interest.

## Publisher’s Note

All claims expressed in this article are solely those of the authors and do not necessarily represent those of their affiliated organizations, or those of the publisher, the editors and the reviewers. Any product that may be evaluated in this article, or claim that may be made by its manufacturer, is not guaranteed or endorsed by the publisher.

## Abbreviations

DECT, Dual-energy computed tomography; OTSCC, Oral tongue squamous cell carcinoma; OSCC, Oral squamous cell carcinoma; PNI, Perineural invasion; λ_Hu,_ Spectral Hounsfield unit curve; nIC, Normalized iodine concentration; nZ_eff,_ Normalized effective atomic number; nRho, Normalized electron density; AP, Arterial phase; VP, Venous phase; DCE, Dynamic contrast-enhanced; DKI, Diffusion kurtosis imaging; HU, Hounsfield unit; AJCC, American Joint Committee on Cancer; VIF, Variance inflation factors.
