# Supplementary material for: Preoperative Prediction of the Aggressiveness of Oral Tongue Squamous Cell Carcinoma with Quantitative Parameters from Dual-Energy Computed Tomography
Source: Front Oncol. 2022 Jun 23;12:904471. doi: 10.3389/fonc.2022.904471 (PMC9260668; doi:10.3389/fonc.2022.904471)
Supplement: Supplementary file 1 [file Table_1.docx]

**Supplementary materials**

**Table S1**. The inter- and intra-observer intraclass correlation coefficients for quantitative DECT parameters

| Parameters | Inter-observer ICC (95%CI) | Intra-observer ICC (95%CI) |
| --- | --- | --- |
| λ_Hu_ in AP | 0.963 (0.944, 0.975) | 0.978 (0.966, 0.985) |
| nIC in AP | 0.970 (0.955, 0.980) | 0.980 (0.969, 0.986) |
| nZ_eff_ in AP | 0.938 (0.906, 0.959) | 0.975 (0.963, 0.984) |
| nRho in AP | 0.951 (0.926, 0.968) | 0.974 (0.961, 0.983) |
| λ_Hu_ in VP | 0.976 (0.964, 0.984) | 0.987 (0.981, 0.991) |
| nIC in VP | 0.987 (0.980, 0.991) | 0.987 (0.981, 0.991) |
| nZ_eff_ in VP | 0.947 (0.920, 0.965) | 0.970 (0.954, 0.980) |
| nRho in VP | 0.929 (0.893, 0.953) | 0.967 (0.950, 0.978) |

DECT, Dual-energy computed tomography; ICC, intraclass correlation coefficients; CI, confidential interval; λ_Hu_, slope of the spectral Hounsfield unit curve; nIC, normalized iodine concentration; nZ_eff_, normalized effective atomic number; nRho, normalized electron density; AP, arterial phase; VP, venous phase﻿
